# Ostéo-arthrite tuberculeuse tarsienne: à propos d’un cas

**Published:** 2012-04-10

**Authors:** Hicham Yacoubi, Moncef Erraji, Rachid Abdelillah, Najib Abbassi, Najib Abdeljawad, Abdelkrim Daoudi

**Affiliations:** 1 Service d’orthopédie, Centre Hospitalier d’Oujda, Faculté de médecine et de pharmacie d’Oujda, Maroc

**Keywords:** Calcanéus, ostéo-arthrite tuberculeuse, Maroc

## Abstract

Nous rapportons le cas d’une patiente de 45 ans sans antécédents médicaux particuliers, qui a présenté une atteinte inflammatoire du pied gauche, sans notion de traumatisme ni de fièvre, avec apparition secondaire d’une fistule cutanée purulente à la face externe du cou de pied. Les radiographies standards et la Tomodensitométrie de la cheville mettaient en évidence une ostéite calcanéenne avec atteinte articulaire subtalienne. Une biopsie chirurgicale associée à une excision des tissus inflammatoires et nécrotiques et l’ablation du trajet fistuleux, ont été réalisées. L’analyse histologique montrait une image de granulome épithélio-giganto-cellulaire avec une nécrose caséeuse et les prélèvements bactériologiques (retrouvaient *Mycobacterium tuberculosis*. Une chimiothérapie antituberculeuse a été administrée pendant 12 mois. À 24 mois, la patiente ne présentait pas de récidive mais une arthropathie dégénérative secondaire subtalienne. Il nous semble intéressant de rappeler que tout tableau clinique traînant ou toute lésion osseuse suspecte et de présentation atypique doit faire évoquer le diagnostic de tuberculose afin d’éviter des retards de diagnostic. Ceci permet une prise en charge thérapeutique précoce de la pathologie.

## Introduction

La tuberculose ostéo-articulaire représente environ 35% des atteintes extra pulmonaires et environ 1 à 3% de tous les cas de tuberculoses [1, 2]. Tous les os sont susceptibles d’être atteints mais certaines localisations sont rares (atteintes des extrémités moins de 10%) [1].

L’absence de familiarité de certains praticiens avec certaines localisations rares de cette pathologie entraîne souvent des retards importants de diagnostic, d’autant plus que la présentation clinique souvent atypique est insidieuse au début de la maladie [1, 2].

Il nous a semblé important de rappeler par notre observation, l’épidémiologie, la pathogénie, les manifestations cliniques, les méthodes diagnostiques et la prise en charge des atteintes ostéoarticulaires extra rachidiennes de la tuberculose notamment du tarse.

## Patient et observation

Il s’agit d’une patiente âgée de 45 ans, sans antécédents médicaux particuliers, qui s’est présentée dans notre formation pour douleur, gonflement et impotence fonctionnelle du pied gauche avec une fistule purulente à la face externe du talon. Son histoire avait débuté six mois auparavant par un état inflammatoire du pied gauche. Elle avait consulté dans une autre formation hospitalière où le diagnostic de cellulite du pied a été posé et une antibiothérapie à base de fluoroquinolone par voie orale pendant 10 jours avait été prescrite. L’évolution clinique n’étant pas favorable, avec persistance de la douleur et de l’impotence fonctionnelle.

**Figure 1:**
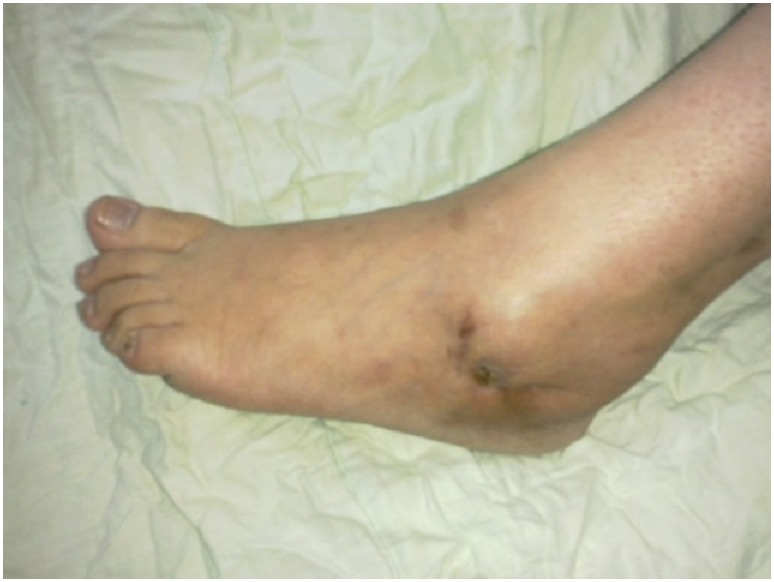
Trajet fistuleux purulent à la face externe du talon

Devant l’apparition d’une fistule cutanée ramenant du pus, la patiente s’est présentée dans notre service pour avis secondaire. La patiente ne rapportait aucun épisode de traumatisme ni de fièvre. Elle présentait à l’examen clinique un pied oedématié inflammatoire avec la présence d’un trajet fistuleux purulent à la face externe du talon (Figure 1).

Les examens biologiques révélaient un léger syndrome inflammatoire avec vitesse de sédimentation à 40 mm à la 1^ère^ heure et une CRP à 7 mg/l. Il n’y avait pas d’hyperleucocytose.

**Figure 2:**
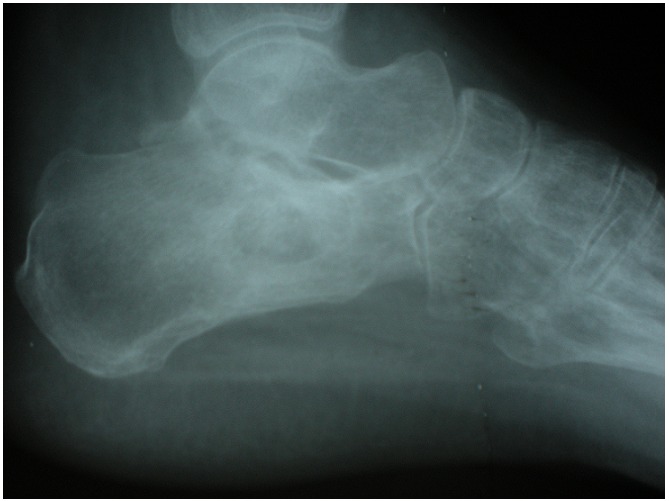
Lacune intra-osseuse du calcanéus

**Figure 3:**
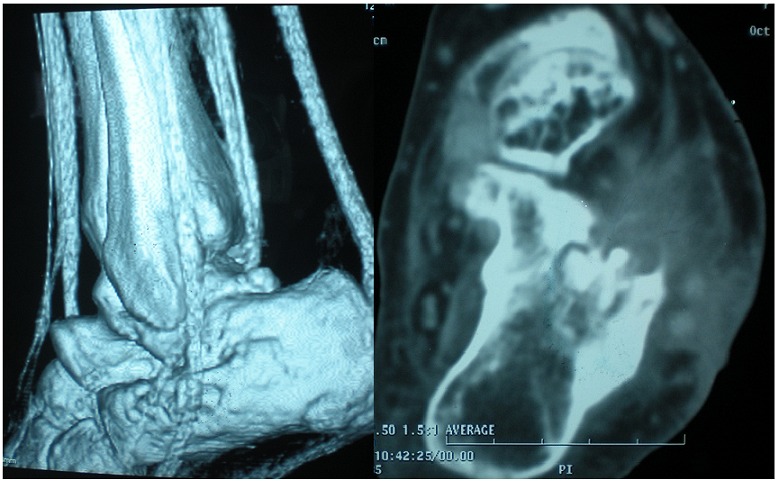
TDM de l’arrière pied montrant la présence d’un séquestre central au sein de la lacune avec des tendons fibulaires intacts

L’intradermoréaction était positive et la sérologie VIH négative. La radiographie standard montrait une lacune intra-osseuse entourée d’hyperostose au versant antéro-supérieur du calcanéus (Figure 2). La radiographie pulmonaire était normale. La TDM avec reconstruction 3D de la cheville et de l’arrière pied a confirmée l’atteinte ostéo-articulaire et a objectivé la présence d’un séquestre central au sein de la lacune. Les tendons fibulaires étaient dans leur partie distale au contact de l’infiltrat hétérogène décrit mais continus et sans signe de ténosynovite extensive (Figure 3).

**Figure 4:**
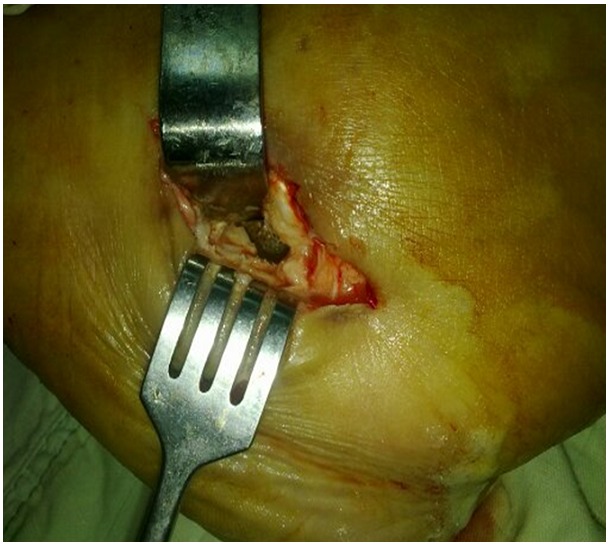
Abord latéral du talon emportant le trajet fistuleux et abordant la géode intra-osseuse

Un parage chirurgical, avec excision des tissus inflammatoires et nécrotiques et résection du trajet fistuleux, a été réalisé. L’abord a été latéral, emportant le trajet fistuleux et abordant la géode visualisée sur l’imagerie préopératoire sous contrôle de l’amplificateur de brillance ayant permis une biopsie osseuse et une necrosectomie avec séquestrectomie. Un nettoyage articulaire sous pression au sérum physiologique a été pratiqué (Figure 4).

**Figure 5:**
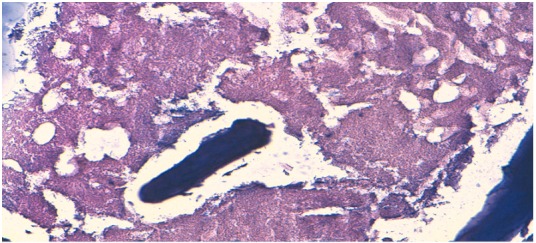
Aspect histologique de la lésion

**Figure 6:**
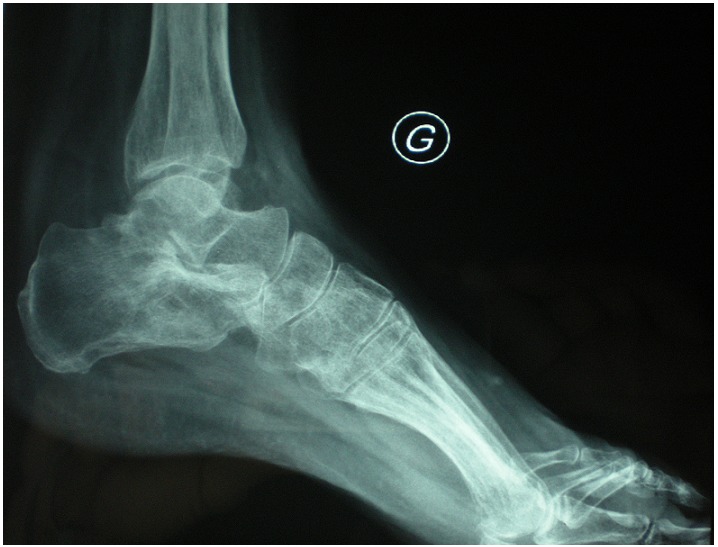
Disparition de la lacune osseuse à 12 mois avec chondrolyse dégénérative de l’articulation subtalienne postérieure

Une immobilisation par attelle postérieure a été mise en place pendant 15 jours. L’examen histologique a mis en évidence de nombreuses cellules inflammatoires formant de façon focale des granulomes à cellules épithélioïdes et géantes, associés à de larges plages de nécrose caséeuse (Figure 5). Cette image a fait évoquer le diagnostic d’ostéite tuberculeuse et a été confirmée par les prélèvements bactériologiques (*Mycobacterium tuberculosis*). La patiente a été mise sous quadrichimiothrérapie antituberculeuse pendant 2 mois suivis d’une bichimiothérapie pour une durée totale de 12 mois. L’évolution fut lentement favorable sur le plan clinique avec disparition des phénomènes inflammatoires locaux et cicatrisation de la plaie opératoire. Sur le plan radiologique, un an après l’instauration de la chimiothérapie antituberculeuse, il y avait une disparition de la lacune osseuse mais la patiente accusait des douleurs mécaniques en rapport avec une chondrolyse dégénérative progressive de l’articulation subtalienne postérieure (Figure 6).

## Discussion

La tuberculose osseuse de la cheville reste très rare. Le talus et le calcanéus restent les os les plus touchés [1, 3]. L’absence d’expérience des praticiens avec ces localisations ostéo-articulaires rares même dans un pays endémique comme le Maroc et les manifestations cliniques atypiques expliquent le retard diagnostique pouvant aller de quelques semaines à plusieurs mois [1, 3].

L’atteinte de la cheville se manifeste par la douleur, le gonflement et l’impotence fonctionnelle. À un stade tardif, notre patiente a présenté une fistulisation à la peau [3]. Le diagnostic repose sur un faisceau d’arguments cliniques et radiologiques. Devant des manifestations chroniques de type douleur et gonflement spontanés de la cheville où le diagnostic reste non précisé et dont la réponse aux anti-inflammatoires reste faible, il faut évoquer le diagnostic de tuberculose. La radiographie osseuse standard est aspécifique. Au début, la tuméfaction des tissus mous et l’ostéopénie peuvent être présentes avant les signes tardifs de destruction osseuse [5]. L’intradermoréaction à la tuberculine (IDR) est positive dans 90% des cas de patients immunocompétents mais sa négativité n’exclut pas le diagnostic [1]. La radiographie du thorax n’est pas d’une grande spécificité car plus de 80% des patients n’ont pas de tuberculose active concomitante [1].

L’imagerie par résonance magnétique (IRM) est sensible dès le début de l’infection grâce à son contraste qui permet de distinguer nettement les zones osseuses infectées des zones saines, mais également par sa capacité à montrer l’extension de l’infection aux tissus mous et aux articulations avoisinantes de l’os atteint [3]. La TDM est en retard par rapport à l’IRM pour la détection de l’atteinte osseuse et bien moins performante pour l’atteinte de tissus mous [5].

La cheville est une articulation superficielle. Elle offre les possibilités d’une biopsie guidée par TDM ou chirurgicale dite à ciel ouvert dans le but de réaliser une analyse histologique et bactériologique est indispensable pour confirmer le diagnostic. La biopsie guidée par un repérage tomodensitométrique est une technique simple et répétitive pouvant se faire sous anesthésie locale. Elle met en évidence un granulome dans 73% des cas, l’examen direct est positif dans 64% des cas, et la culture est positive dans 83% des cas [6]. La biopsie chirurgicale, quant à elle, offre l’avantage de fournir un matériel abondant et donne des résultats positifs dans presque 90% des cas, comme l’ont montré Lemaître et al. [7].

L’image histologique classique est celle d’un granulome épithélio-giganto-cellulaire avec ou sans nécrose caséeuse [7].

Le traitement fait appel à une quadrichimiothérapie comprenant la rifampicine (DCI) (600 mg/j), l’isoniazide (DCI) (5 mg/kg), la pyrazinamide (DCI) (1,5 à 2 g/j) et l’ethambutol (DCI) (15 mg/kg) pendant au moins deux mois et il faut poursuivre une bithérapie par INH et RMP. Dans notre pratique la durée minimale de traitement est de neuf mois.

La principale cause d’échec du traitement est la mauvaise observance thérapeutique, qui favorise les échecs primaires, les rechutes et l’acquisition de résistances secondaires. Cette constatation a été à l’origine de l’idée anglo-saxonne de la directly observed therapy (DOT) qui repose sur la fourniture régulière des antibiotiques au patient et sur le contrôle de sa prise par une tierce personne (infirmière ou un travailleur social). Cette stratégie a été recommandée par l’OMS et est appliquée dans notre pays.

Le traitement orthopédique en phase aiguë consiste à mettre en décharge stricte l’articulation de la cheville avec un appareillage d’immobilisation dans une position fonctionnelle, jusqu’à la disparition des phénomènes inflammatoires. Nous avons délaissé dans notre service toute immobilisation articulaire prolongée, source d’une ankylose spontanée et nous démarrons une rééducation concomitamment au traitement médical.

Le traitement chirurgical secondaire interviendra dans le cadre des corrections de déformation séquellaire importante et douloureuse. Au niveau du pied et de la cheville, c’est principalement les gestes d’arthrodèse complémentaire qui seront proposés sur les lésions dégénératives post-infectieuses.

## Conclusion

Le traitement de la tuberculose ostéoarticulaire nécessite la collaboration entre médecin, bactériologiste et chirurgien. L’observance thérapeutique est une nécessité absolue. Différentes recommandations internationales ont été motivées par l’augmentation du taux de résistance des BK aux antituberculeux. La prise en charge des problèmes sociaux et professionnels est souvent nécessaire chez les adultes jeunes. La tuberculose est une maladie à déclaration obligatoire.
